# Identification of QTLs for resistance to 10 pathotypes of *Plasmodiophora brassicae* in *Brassica oleracea* cultivar ECD11 through genotyping-by-sequencing

**DOI:** 10.1007/s00122-023-04483-y

**Published:** 2023-11-20

**Authors:** Md. Masud Karim, Fengqun Yu

**Affiliations:** Saskatoon Research and Development Centre, Agriculture and Agri-Food Canada, 107 Science Place, Saskatoon, SK S7N 0X2 Canada

## Abstract

**Key message:**

Two major quantitative trait loci (QTLs) and five minor QTLs for 10 pathotypes were identified on chromosomes C01, C03, C04 and C08 through genotyping-by-sequencing from *Brassica oleracea*.

**Abstract:**

Clubroot caused by *Plasmodiophora brassicae* is an important disease in brassica crops. Managing clubroot disease of canola on the Canadian prairie is challenging due to the continuous emergence of new pathotypes. *Brassica oleracea* is considered a major source of quantitative resistance to clubroot. Genotyping-by-sequencing (GBS) was performed in the parental lines; T010000DH3 (susceptible), ECD11 (resistant) and 124 BC_1_ plants. A total of 4769 high-quality polymorphic SNP loci were obtained and distributed on 9 chromosomes of *B. oleracea*. Evaluation of 124 BC_1_S_1_ lines for resistance to 10 pathotypes: 3A, 2B, 5C, 3D, 5G, 3H, 8J, 5K, 5L and 3O of *P. brassicae*, was carried out. Seven QTLs, 5 originating from ECD11 and 2 from T010000DH3, were detected. One major QTL designated as *Rcr_C03-1* on C03 contributed 16.0–65.6% of phenotypic variation explained (PVE) for 8 pathotypes: 2B, 5C, 5G, 3H, 8J, 5K, 5L and 3O. Another major QTL designated as *Rcr_C08-1* on C08 contributed 8.3 and 23.5% PVE for resistance to 8J and 5K, respectively. Five minor QTLs designated as *Rcr_C01-1, Rcr_C03-2, Rcr_C03-3, Rcr_C04-1* and *Rcr_C08-2* were detected on chromosomes C01, C03, C04 and C08 that contributed 8.3–23.5% PVE for 5 pathotypes each of 3A, 2B, 3D, 8J and 5K. There were 1, 10 and 4 genes encoding TIR-NBS-LRR/CC-NBS-LRR class disease resistance proteins in the *Rcr_C01-1*, *Rcr_C03-1* and *Rcr_C08-1* flanking regions. The syntenic regions of the two major QTLs *Rcr_C03-1* and *Rcr_C08-1* in the *B. rapa* genome ‘Chiifu’ were searched.

**Supplementary Information:**

The online version contains supplementary material available at 10.1007/s00122-023-04483-y.

## Introduction

Brassica species are grown as source of oil, vegetable, condiment, and fodder worldwide. The ‘Triangle of U’ (Morinaga 1934; Nagaharu and Nagaharu 1935) revealed relationships among the main species of Brassica genus, which makes more interesting of these species to the researchers for genomic utilization among the species. *B. napus* (AACC; *n* = 19), *B. juncea* (AABB; *n* = 18) and *B. carinata* (BBCC; *n* = 17) are amphidiploid species resulting from hybridization between pairs of the diploid species: *B. rapa* (AA; *n* = 10), *B. nigra* (BB; *n* = 8) and *B. oleracea* (CC; *n* = 9).

Clubroot, caused by *Plasmodiophora brassicae* Woronin, a soil-borne obligate biotrophic protist, is an important disease in brassica crops worldwide. The pathogen causes club or spindle shape gall formation on roots, which hampers nutrients and water uptake for plants growth. The disease has been reported in more than 60 countries, causing about 10–15% yield loss globally (Dixon 2009). The disease not only caused yield loss but also affected the quality of the crops (Engqvist 1994; Pageau et al. 2006). Some practices such as liming and early spring seeding can reduce clubroot levels but is not economical for large-scale canola production (Voorrips et al. 1995; Gossen et al. 2012, Donald et al. 2014; Hwang et al. 2014). Utilization of clubroot resistant (CR) cultivars is the most effective way to control the disease (Peng et al. 2014; Rahman et al. 2014). However, repeated utilization of the same CR source is not a good strategy as it provides opportunity to minor pathotypes to become predominant pathotypes (Sedaghatkish et al. 2019; Cao et al. 2020). In Canada, breeding companies released CR varieties in 2009–2010 after identifying clubroot disease in canola fields in 2003 (Tewari et al. 2005). Most CR canola cultivars probably were developed by using a CR gene from the oilseed rape ‘Mendel’ (Fredua-Agyeman et al. 2018), which became susceptible just after 4 years in 2013 due to emergence of new virulent pathotype 5X (Strelkov et al. 2016). This incidence led to closely focus on pathogen populations in Canadian canola fields. As a result, > 30 pathotypes have been classified based on the Canadian Clubroot Differential (CCD) set (Strelkov et al. 2018; Hollman et al. 2021). In addition to the most prevalent original pathotype 3H, current predominant new pathotypes 3A, 3D (Dakouri et al. 2021) and other pathotypes are also needed to consider to find new resistant sources. Along with crop rotation, gene pyramiding from different sources is considered an effective strategy for successful control of clubroot disease caused by different pathotypes of the pathogen.

As resistance sources to clubroot in *B. napus* (AACC) are very limited, researchers have searched resistance in its diploid progenitor species *B. rapa* (AA) and *B. oleracea* (CC). Genes can be transferred from *B. rapa* (AA) and *B. oleracea* (CC) to *B. napus* either interspecific breeding or developing new re-synthesized *B. napus*. CR in *B. rapa* was usually controlled by major genes, while CR in *B. oleracea* was mostly reported as quantitative trait loci (QTL), polygenic control. Although polygenic resistance is considered more durable and can be race non-specific (Diederichsen et al. 2009), resistance sources in *B. oleracea* are very limited (Dias et al. 1993; Manzanares-Dauleux et al. 2000; Voorrips 1995; Diederichsen et al. 2009). Cabbage (*B. oleracea* var. *capitata*) cultivar ‘Badger Shipper’ (ECD11) showed resistance to 15 pathotypes out of 17 pathotypes in the CCD Set (Strelkov et al. 2018), which can be very useful as race non-specific CR source.

Identification of QTLs for resistance to clubroot has been performed in *B. oleracea*. Four QTLs, *qCRc4-1* on C04, *qCRc7-*2, *qCRc7-3*, and *qCRc7-4* on C07 were identified through QTL-Seq for resistance to race 4 of *P. brassicae* in cabbage breeding line GZ87 (Ce et al. 2021). Another two QTLs, *DIC.I-1* and *DIC.II-1*, were identified on C08 from the same *B. oleracea* inbred line GZ87 against race 4 (Peng et al. 2018). Ten QTLs; *PbC4.1*, *PbC6*, *PbC7.1*, *PbC7.2*, *PbC8*, *PbC9.1*, *PbC3*, *PbC4.2*, *PbC7.3* and *PbC9.2* from six C-genome chromosomes; C03, C04, C06, C07, C08 and C09 were identified through association mapping against pathotype 3A and 5X-LG2 (Farid et al. 2020). Two QTLs, *CRQTL-GN_1* and *CRQTL-GN_2* on C02 and C03 for resistance to race 9, and one QTL, *CRQTL-YC* on C03 for resistance race 2 were identified (Lee et al. 2016). One major QTL, *pb-Bo(Anju)1* on C02, four minor QTLs *pb-Bo(Anju)2* on C02, *pb-Bo(Anju)3* on C03, *pb-Bo(Anju)4* on C07 and *pb-Bo(GC)1* on C05 were derived from resistant line Anju and susceptible line GC (Nagaoka et al. 2010). A total of nine QTLs, *Pb-Bo1*, *Pb-Bo2*, *Pb-Bo3*, *Pb-Bo4*, *Pb-Bo5a*, *Pb-Bo5b*, *Pb-Bo8*, *Pb-Bo9a* and *Pb-Bo9b*, were identified from a selected line (C10) of a French kale landrace (*B. oleracea* var. acephala) for clubroot resistance on C01–05, C08–09 against five isolates, Ms6 and eH (pathotype P1), K92 (pathotype P2), K92-16 (pathotype P4), Pb137-522 (pathotype P7) of *P. brassicae*. Two to five QTLs were detected depending on the isolates. The *Pb-Bo1* was detected in all isolates and explained 20.7–80.7% of the phenotypic variation (Rocherieux et al. 2004). A QTL was identified on C03 from a inbreed kale line K269 against pathotype 1 and 3 (Moriguchi et al. 1999). Two QTLs, *pb-3* and *pb-4*, were identified from the German landrace ‘Bindsachsener’ on C03 and C01 against a field isolate characterized as ECD 16/3/30 (Voorrips et al. 1997). Only one major dominant CR gene (*Rcr7*) was identified from chromosome C7 originating from A-genome of *B. rapa* (Dakouri et al. 2018).

Although no CR gene has been cloned from C-genome, three CR genes *CRa, Crr1* and *CRb*^kato^ were cloned from A-genome, which encode toll-interleukin-1 receptor, nucleotide binding site and leucine-rich repeat (TIR-NBS-LRR) proteins (Ueno et al. 2012; Hatakeyama et al. 2013, 2017). Resistant genes encoded NBS-LRR protein families can be subdivided according to their functional domain as TIR domain containing (TNL) and coiled-coil (CC) domain containing (CNL) subfamilies (McHale et al. 2006). NBS-LRR-related disease resistance is effective against obligate and hemi-biotrophic pathogens (Glazebrook et al. 2005). Although TNL protein encoding genes are reported for CR resistance, no CR gene encoding CNL protein has been reported.

In this study, a BC_1_/BC_1_S_1_ mapping population was developed using resistant cabbage cultivar ECD11 crossed with clubroot-susceptible doubled haploid (DH) line T010000DH3. The objectives of the current study were: 1) to characterize the genome-wide DNA variants in the BC_1_ lines through genotyping-by-sequencing (GBS); 2) to identify QTLs associated with resistance to 10 pathotypes of *P. brassicae* characterized with the CCD set; and 3) to identify possible candidate genes for each QTL.

## Materials and methods

### Plant materials

Seeds of resistant cultivar ECD11 and susceptible DH line T010000DH3 derived from Chinese kale (*B. oleracea*) cultivar ‘TO1434’ were provided by Dr. G. R. Dixon (The University of Warwick, Wellesbourne, Warwick, UK) and Dr. I. Parkin (Saskatoon Research and Development Centre, Saskatoon, SK, Canada), respectively. ECD11 was crossed to T010000DH3 (pollen donor) to produce F_1_ progeny. Backcross with T010000DH3 (recurrent parent) was performed to produce the BC_1_ population. In total, 124 BC_1_ plants were used for GBS and self-pollinated to produce a BC_1_S_1_ population consisting of 124 lines for inoculation testing at the Saskatoon Research and Development Centre.

### Evaluation of plants for resistance to clubroot

Strains of *P. brassica*e collected from canola fields in Alberta were provided by Dr. S.E. Strelkov at the University of Alberta, Canada. The inoculum preparation method used in the study was as described by Suwabe et al. (2003). Seedlings of a susceptible cultivar were inoculated and maintained under controlled conditions. Clubbed roots were harvested from infected plants after 5–6 weeks and stored at − 20 °C. Fresh inoculum of each pathotype was prepared after softening about 5 g of frozen club in a small amount of distilled water for 1–2 h. The material was homogenized in a blender for 2 min and strained through 2–3 layers of nylon mesh cloth. The resulting spore suspension was diluted with deionized water to produce a final concentration of 1 × 10^7^ resting spores mL^−1^. Plants were tested for resistance to 10 pathotypes of *P. brassicae* (strain F.3–14 for pathotype 3A, F.183–14 for 2B, F.175–14 for 5C, F.1–14 for 3D, CDCS for 5G, P. 41–14 for 3H, F.12–15 for 8J, F.10–15 for 5K, CDCN#2 for 5L and F.381–16 for 3O). The inoculation method used in the current study was as described by Yu et al. (2021). Seeds of the BC_1_S_1_ population were sown into Sunshine #3 soilless mix (Sun Gro Horticulture Canada Ltd.; Seba Beach, AB) with Osmocote (Everris NA Inc.; Dublin, OH, USA) in 32-pots inserts held by trays (The HC Companies; Twinsburg, OH, USA). Adequate amount of water was added to each tray to soak the soilless mix overnight. Similar experimental design as described by Yu et al. (2017) was used in this study. Ten to twelve seeds were sown in each pot/line, which were inoculated with 15 ml of inoculum (1 × 10^7^ spores/ml) after seven days of sowing. The tray holding pot was covered with a dome for two weeks. The inoculated plants were grown in a growth chamber set at 22/18 °C day/night temperature with a 16 h photoperiod. The pots were always kept moist by adding adequate amount of water whenever necessary. Leaves of the plants were pruned every alternate days after two weeks of seeding. Watering was stopped at 2 days before ratting clubroot. The *B. rapa* var. *pekinensis* ‘Granaat’ ECD05 (susceptible to all 10 pathotypes used in this study) and the parental lines (ECD11 and T010000DH3) were included as controls.

Six weeks after inoculation, each plant was rated for clubroot symptoms using a 0–3 scale (Kuginuki et al. 1999), where 0 = no symptoms, 1 = a few small clubs, 2 = moderate clubbing, and 3 = severe clubbing (Fig. S1). A disease severity index (DSI) was calculated for each line using the method of Horiuchi and Hori (1980):

$${\text{DSI}} = \frac{{\sum {({\text{rating}}\;{\text{class}})} \times (\# \;{\text{plants}}\;{\text{in}}\;{\text{rating}}\;{\text{class}})}}{{{\text{total}}\;\# \;{\text{plants}}\;{\text{in}}\;{\text{treatment}} \times 3}} \times 100$$

Pearson correlation coefficient was calculated with disease severity indexes (DSIs) of 124 BC_1_*S*_1_ populations.

The *F*_1_ (T010000DH3 × ECD11) also included in the inoculation test for all the ten pathotypes in this study. Reciprocal F_1_ (ECD11 × T010000DH3) were tested with only 3 pathotypes 3A, 2B and 3D as only few seeds from the cross were obtained, so it was not possible to test all pathotypes. The experiment was repeated twice. Each of the repetitions provided a similar result in most cases. For those lines with inconsistent results, the higher DSI among the two repetitions of the assessment was considered to be more accurate and was used to characterize the resistance response of the line.

### GBS of the parental lines and 124 BC_1_ plants

Young leaves of the 124 BC_1_ plants and two replications of parental lines were collected and freeze-dried in a Freezone 6 dryer (Labconco Corp, Kansas City, MO) for 48 h and grinded using the Mixture Mills 300 (Retsch Inc., Newtown, PA). DNA was extracted using DNeasy 96 Plant Kit (Qiagen, Toronto, ON, Canada) following DNeasy Plant Handbook from QIAGEN, quantified using a NanoVue Plus spectrophotometer (GE Healthcare, Piscataway, NJ), diluted to 10 ng/μl and kept at −20 °C until subsequent use for sequencing and genotyping. In total, 128 DNA samples were prepared for sequencing. GBS library generation, PstI/MspI for Illumina with size selection were done in Institute of integrative biology and systems (Université Laval, Québec, Canada). Illumina library QC and Illumina HiSeq2500 PE125 sequencing were performed at Genome Quebec (Montreal, Canada). Two replications of parental lines were performed to increase the sequencing depth, to provide a more accurate call of the genotype at each SNP site in the BC_1_ population.

### Alignment of GBS short reads into *B. oleracea* reference genome sequence and SNP calling

The reference genome sequence of DH line ‘D134’ (cabbage; *B. oleracea* var. *capitata*) generated by third-generation sequencing technology (Lv et al. 2020) and downloaded from https://db.cngb.org/search/?q=CNP0000469 was used for alignment and data analysis. Short reads from each of the 124 BC_1_ samples, combined two replicates of parental lines ECD11 and T010000DH3, were aligned to the reference genome sequence. GBS-SNP-CROP pipeline v3.0 (Melo et al. 2016) was used to get SNP panel using Linux platform of AAFC Biocluster, following steps were performed: (1) parse the raw reads, (2) trim based on quality and adaptors, (3) demultiplex, (4) align with BWA-MEM and process with samtools, (5) parse mpileup outputs and produce the variant discovery matrix, (6) filter variants and call genotypes, and (7) create input files for downstream analyses. The trimming of the reads was performed using trimmomatic-0.39 (Bolger et al. 2014) prior to the read mapping. Trimming parameters were used as follows: data type = paired-end, number of threads = 10, phred quality score = 33, Trimmomatic ILLUMINACLIP string = TruSeq3-PE.fa:2:30:10:8:true, Trimmomatic LEADING value = 30, Trimmomatic SLIDINGWINDOW value 4:30, Trimmomatic TRAILING value = 30, Trimmomatic MINLEN value = 32. Identification of DNA variants (SNPs and InDels) in the DNA sequences each BC_1_ sample relative to the reference genome sequence was performed using the GBS-SNP-CROP pipeline v3.0, but only SNPs were used for further study. SNPs were filtered with the following parameters: mnHoDepth0 = 5, mnHoDepth1 = 20, mnHetDepth = 3, altStrength = 0.96, mnAlleleRatio = 0.25, mnCall = 0.75, mnAvgDepth = 3 and mxAvgDepth = 200. SNP panel from the 124 BC_1_ plants along with the parental lines ECD11 and T010000DH3 were used to get polymorphic sites in TASSEL5 (Bradbury et al. 2007). KNNimp imputation was done to recover missing data. Data were filtered with minimum count 5, minimum frequency 0.1 (10%), maximum frequency 1.0 (100%). SNP alleles from the susceptible parent (T010000DH3) were scored as ‘*a*’, resistant parent (ECD11) were scored as ‘*b*’ and heterozygous allele as ‘*h*’. As the parent T010000DH3 is a DH line, all SNP sites should theoretically be homozygous (a). Excel sort function was used to filter out allele ‘h’ from the DH line and SNP with higher missing sites.

### Construction of linkage map and QTL mapping

To remove redundant markers, another two-step filtering was performed with JoinMap and the BIN function with ICIMapping. The high-quality polymorphic SNPs were further analyzed using JoinMap 4.1 (Van Ooijen 2011). Maximum likelihood in Kosambi’s model with a minimum logarithm of the odds (LOD) values of 10 was used to determine marker orders and positions in the genetic map. Only SNP sites that could be assigned into the 9 chromosomes of the C-genome sequence at LOD scores of 10.0 were kept. The set of filtered SNP sites obtained from JoinMap4.1 was used for binning of redundant markers, construction of linkage map, and mapping of QTLs for resistance to clubroot using the QTL IciMapping Inclusive Composite Interval Mapping (ICIM) method (Meng et al. 2015). Mapchart 2.1 (Voorrips 2002) was used to draw a linkage map based on the genetic location determined with QTL IciMapping. The LOD score threshold was set using a 1,000-permutation test with a Type I error of 0.05 for QTL declaration. The QTL effects were estimated as phenotypic variation explained (PVE) and additive (Add) values by each QTL.

### Identification of potential candidate genes in the flanking region of QTL mapping

Coding sequences (CDS) of the genes in the identified QTL flanking regions were extracted from the reference genome sequence of *B. oleracea* ‘D134’ to perform gene annotation with Blast2GO (Conesa et al. 2005). Genes related to disease resistance and defense responses were identified from Blast2GO description. CDS of the disease resistance genes identified from Blast2GO were used for blast search at www.arabidopsis.org to find Arabidopsis homolog genes and the class of disease resistance proteins.

### Search for A-genome syntenic regions of identified C-genome QTLs

The *B. rapa* reference genome sequence v3.0 (Zhang et al. 2018) downloaded from http://brassicadb.org/brad/downloadOverview.php were used to find C-genome synteny of identified QTL. Homolog of *B. oleracea* disease resistance genes to *B. rapa* and Arabidopsis genes were identified by blast search in http://brassicadb.cn/#/BLAST/ and http://www.arabidopsis.org/, respectively.

## Results

### Resistance to clubroot in the *B. oleracea* parental lines, F_1_ and BC_1_S_1_ population

**Fig. 1:**
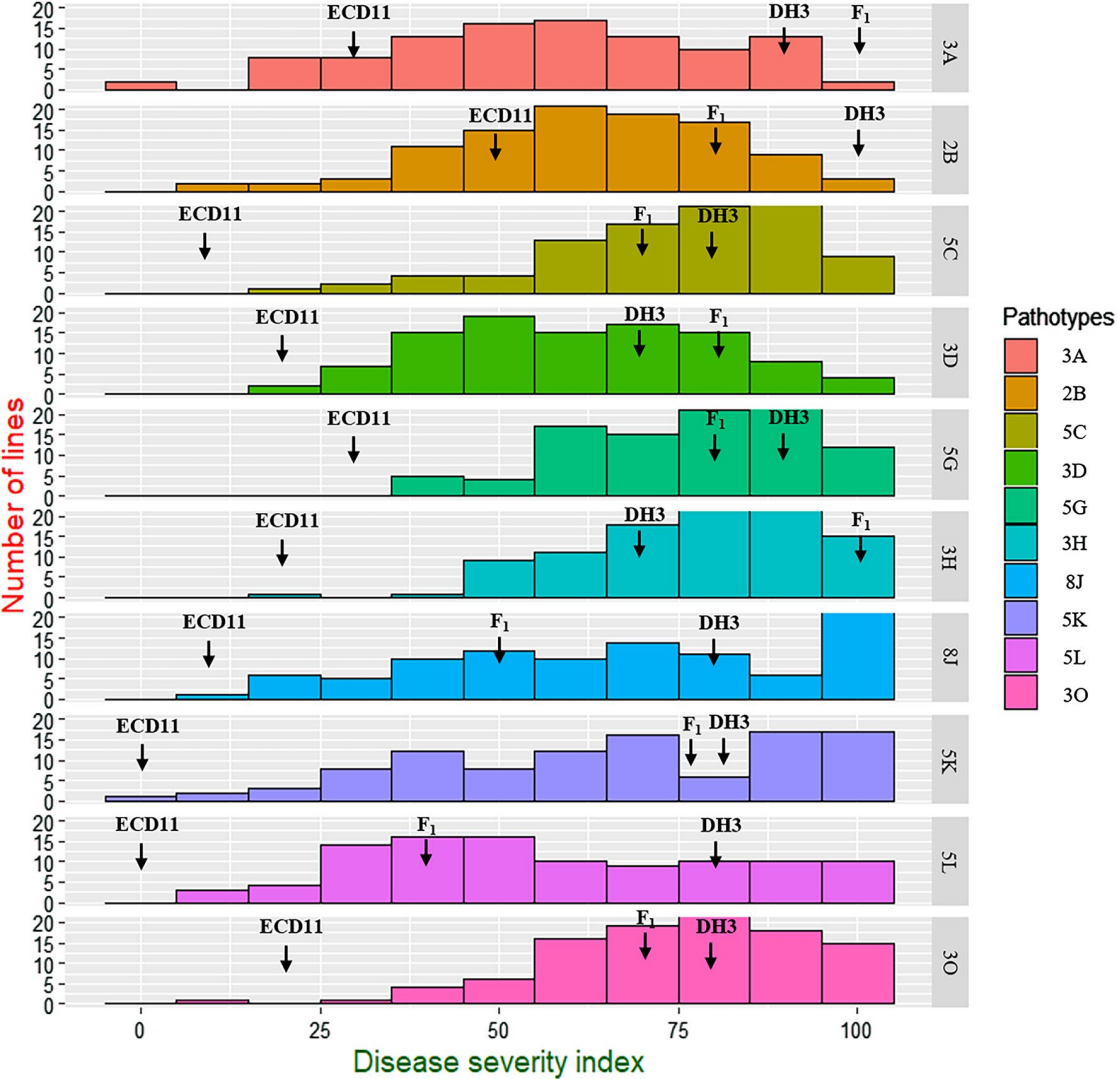
Distribution of disease severity indexes (DSIs) of 124 BC_1_S_1_ population derived from DH3 × (DH3 × ECD11) against 10 pathotypes of *Plasmodiophora brassicae*. Arrows pointed the position of parental and F_1_ plants

The clubroot reaction in the parental lines (ECD011 and T010000DH3), F_1_, controls and BC_1_ population was assessed against 10 pathotypes; 3A, 2B, 5C, 3D, 5G, 3H, 8J, 5K, 5L and 3O as classified on the CCD set (Strelkov et al. 2018) (Fig. 1, Supplementary Table S1). ECD11 was highly or moderately resistant to all pathotypes (0–47.2% DSI), T010000DH3 was susceptible (66.7–94.4% DSI) for the ten pathotypes. The susceptible control ECD05 was highly susceptible to all the pathotypes (100% DSI). The F_1_ plants from the interspecific crosses T010000DH3 × ECD11 were highly susceptible (66.7–100% DSI) to 8 pathotypes; 3A, 2B, 5C, 3D, 5G, 3H, 5K and 3O, moderately resistant to 8J and 5L (33.3–50.0% DSI) (Supplementary Table S1). The frequency distribution in the BC_1_S_1_ population for resistance to all pathotypes showed continuous segregation patterns (Fig. 1). More BC_1_S_1_ lines with DSI distributed at 50–70% for pathotypes 3A, 2B, 3D, 70–90% for pathotypes 5C, 5G, 3H and 3O, 30–50% for 5L, 90–100% for 5K and 100% for 8J. Correlation coefficients among the disease severity index values for the 10 pathotypes ranged from 0.24 to 0.77, but all were significant at *P* < 0.01 except 5K at *P* < 0.05 (Table 1).

**Table 1:** Correlation coefficients for clubroot severity after inoculation of the BC_1_S_1_ population derived from BC_1_ of T010000DH3 × (T010000DH3 × ECD011) for resistance to ten pathotypes of *Plasmodiophora brassicae*

	2B	3A	3D	3H	3O	5C	5G	5K	5L	8J
2B	1.00									
3A	0.55**	1.00								
3D	0.32**	0.30**	1.00							
3H	0.36**	0.33**	0.28**	1.00						
3O	0.29**	0.26**	0.29**	0.31**	1.00					
5C	0.29**	0.30**	0.36**	0.50**	0.56**	1.00				
5G	0.37**	0.37**	0.29**	0.39**	0.40**	0.46**	1.00			
5K	0.45**	0.43**	0.24*	0.47**	0.42**	0.53**	0.58**	1.00		
5L	0.40**	0.36**	0.34**	0.55**	0.57**	0.55**	0.52**	0.70**	1.00	
8J	0.47**	0.44**	0.37**	0.57**	0.45**	0.50**	0.58**	0.72**	0.77**	1.00

*Significance level at *P* < 0.05 or **Significance level at *P* < 0.01

### Alignment of GBS short reads into the *Brassica oleracea* reference genome sequence of cabbage DH line ‘D134’

In total 980.0 million (M) short reads were recovered from Illumina HiSeq2500 pair end sequencing from two replicates of parental lines and 124 BC_1_ lines (Supplementary Table S2). About 17.0 M and 15.4 M short reads were obtained from the parental lines ECD11 and T010000DH3, from there 16.1 M (94.7%) and 14.2 M (92.4%) reads were aligned into the *B. oleracea* reference genome sequence. A total 947.6 M short read sequence from 124 BC_1_ lines were identified ranging 0.9–13.9 M/line, average 7.6 M/line. The average number of reads aligned into the reference genome sequence from each line was 6.9 M, ranging 0.8–12.6 M and 84.4–93.9% (Supplementary Table S2).

### Variants calling and polymorphic SNP selection

Total 8092 SNPs from 124 BC_1_ plants were assigned to nine chromosomes of the reference genome sequence. The average number of SNP/chromosome was 899, ranging 646–1129 of the reference genome sequence ‘D134’(Supplementary Table S3). The number of SNPs identified in the population was strongly correlated (*R*^*2*^ = 0.84) with length of chromosomes in the reference genome sequence of ‘D134’ (Supplementary Table S3). Total 7405 and 8011 SNPs from the parental lines ECD11 and T010000DH3 were assigned to the 9 chromosomes (Supplementary Table S3). The number of SNPs identified in the parental lines ECD11 and T010000DH3 was strongly correlated (*R*^*2*^ = 0.82, 0.84) with length of chromosomes in the reference genome sequence (Supplementary Table S3). Total 4,769 polymorphic SNP sites aligned to 9 chromosomes of ‘D134’ (Supplementary Table S3) and the number of polymorphic SNPs identified in the population were also strongly correlated (*R*^*2*^ = 0.71) with length of chromosomes (Supplementary Table S3). Total number SNPs and polymorphic SNPs were also positively correlated (*R*^*2*^ = 0.65) (Supplementary Table S3).

### Construction of linkage map with comparison previously published map

A total of 1087 SNPs were filtered out in JoinMap and 2268 SNPs were filtered out by BIN function in ICIMapping. A genetic map consisting of 1,256.5 cM (Fig. S2) were constructed from the 1,414 non-redundant polymorphic SNP sites (Supplementary Table S3). The average number of SNPs mapped per chromosome was 157.1, ranging 126–185. The length of each chromosome ranged from 117.6 to 193.4 cM with an average length of 139 cM. The SNP interval of each chromosome ranged from 0.7 to 1.2 cM, with an average of 0.9 cM (Supplementary Table S3 and Table S4).

### QTL mapping

**Fig. 2:**
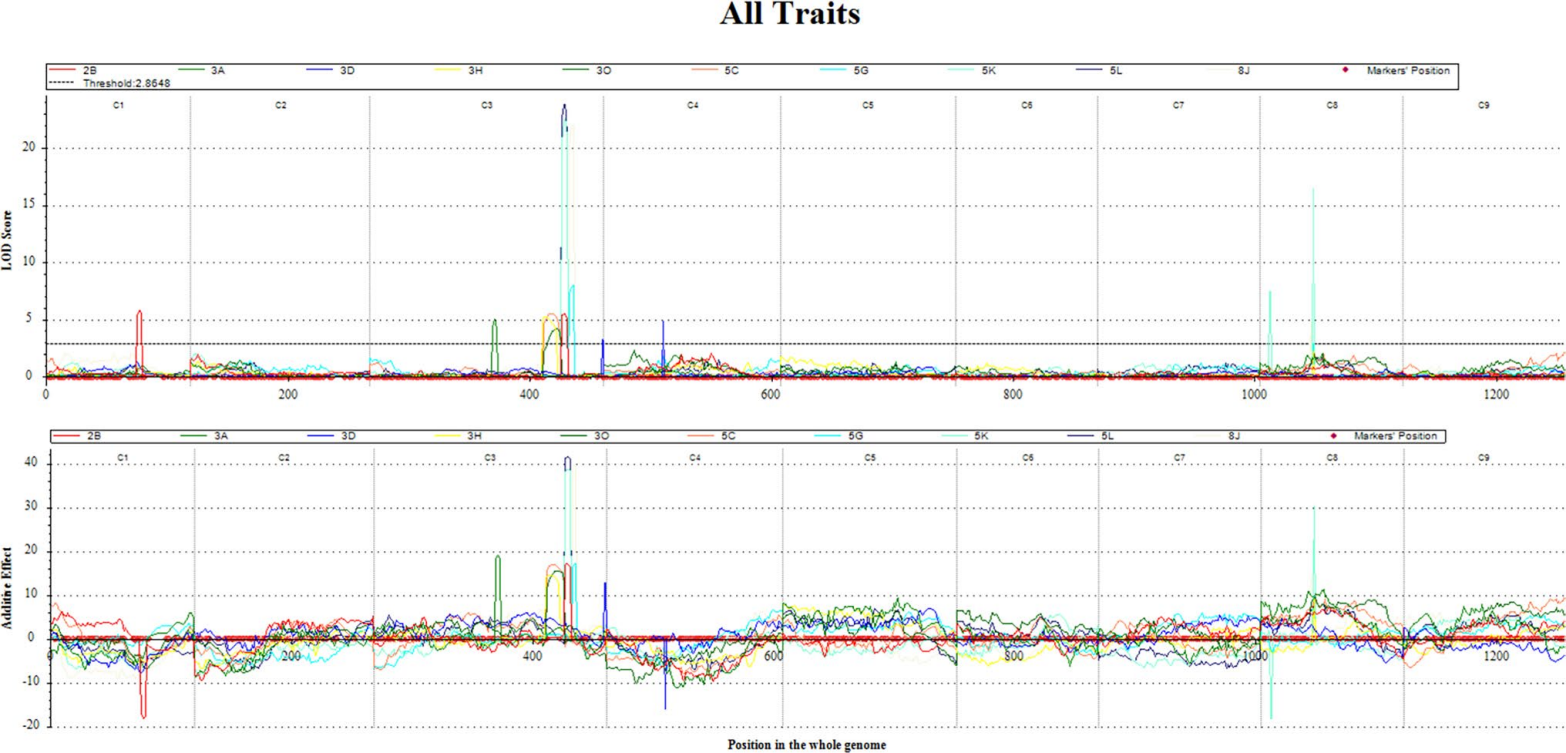
Significant QTL identified on chromosome C01, C03, C04 and C08 for 10 pathotypes of *Plasmodiophora brassicae*; 3A, 2B, 5C, 3D, 5G, 3H, 8J, 5K, 5L and 3O and from *B. oleracea* cultivar ECD11

QTL mapping was performed using the linkage map (Fig. S2) constructed with the 1414 SNP sites and %DSI values for resistance to 10 pathotypes (3A, 2B, 5C, 3D, 5G, 3H,  8J, 5K, 5L and 3O). Seven QTLs were detected on chromosomes C01, C03, C04 and C08 (Fig. 2, Table 2). A single QTL for resistance to pathotype 2B was detected on chromosome C01, designated as *Rcr_C01-1* with the peak between the SNP markers D134_C01_8,398,944 and D134_C01_7,492,399. QTLs for resistance to all the pathotypes were detected on chromosome C03. *Rcr_C03-1* for resistance to 8 pathotypes; 2B, 5C, 5G, 3H, 8J, 5K, 5L and 3O (but not 3A and 3D) was located at the peak between the SNP markers D134_C03_9,211,088 and D134_C03_17,067,147. *Rcr_C03-2* for resistance to pathotype 3D was identified at the peak between the SNP markers D134_C03_585,685 and D134_C03_1,026,778. *Rcr_C03-3* with the peak between the SNP markers D134_C03_35,229,606 and D134_C03 _36,286,546, showed resistance to pathotype 3A. A single QTL was detected on chromosome C04, *Rcr_C04-1* with the peak between the SNP markers D134_C04 _51,280,226 and D134_C04_52,146,319, showed resistance to pathotype 3D. Two QTLs were detected on chromosome C08; QTL *Rcr_C08-1* with the peak between the SNP markers D134_C08_23,354,593 and D134_C08_24,782,998, showed resistance to pathotype 8J and 5K. Another QTL *Rcr_C08-2* with the peak between the SNP markers D134_C08_28,507,471 and D134_C08_29,075,065, showed resistance to pathotype 5K.

**Table 2:** QTL position, interval and phenotypic variation explained (PVE) for resistance of 10 pathotypes of *Plasmodiophora brassicae* in *B. oleracea* cultivar ECD11 using *Brassica oleracea* reference genome sequence of cabbage DH line ‘D134’

Pathotype	Chromosome / QTL	Position	Left marker	Right marker	LOD	PVE (%)	Add	Left CI	Right CI
3A	C03 / *Rcr_C03-3*	103	C03-V3_36,286,546	C03-V3_35,229,606	5.0	18.7	19.0	100.5	105.5
2B	C01 / *Rcr_C01-1*	77	C01-V3_8,398,944	C01-V3_7,492,399	5.8	17.4	18.1	74.5	79.5
	C03 / *Rcr_C03-1*	160	C03-V3_9,689,022	C03-V3_10,341,716	5.5	16.0	17.1	158.5	163.5
5C	C03 / *Rcr_C03-1*	150	C03-V3_17,067,147	C03-V3_9,689,084	5.5	22.2	16.9	143.5	156.5
3D	C03 / *Rcr_C03-2*	192	C03-V3_1,026,778	C03-V3_585,685	3.2	10.2	12.8	191.5	193.0
	C04 / *Rcr_C04-1*	49	C04-V3_52,146,319	C04-V3_51,280,226	4.8	16.0	-6.1	48.5	49.5
5G	C03 / *Rcr_C03-1*	168	C03-V3_13,163,848	C03-V3_9,211,088	8.0	28.6	17.1	164.5	168.5
3H	C03 / *Rcr_C03-1*	145	C03-V3_17,067,147	C03-V3_9,689,084	5.2	20.2	14.3	142.5	153.5
8J	C03 / *Rcr_C03-1*	168	C03-V3_13,163,848	C03-V3_9,211,088	22.1	57.7	39.4	165.5	168.5
	C08 / *Rcr_C08-1*	42	C08-V3_24,782,998	C08-V3_24,249,890	4.7	8.3	14.7	41.5	42.5
5K	C03 / *Rcr_C03-1*	161	C03-V3_10,341,716	C03-V3_12,650,131	22.5	36.9	38.6	159.5	162.5
	C08 / *Rcr_C08-2*	8	C08-V3_29,075,065	C08-V3_28,507,471	7.4	8.5	-8.2	7.5	9.5
	C08 / *Rcr_C08-1*	44	C08-V3_23,699,110	C08-V3_23,354,593	16.4	23.5	30.3	43.5	44.5
5L	C03 / *Rcr_C03-1*	161	C03-V3_10,341,716	C03-V3_12,650,131	23.8	65.6	41.4	159.5	162.5
3O	C03 / *Rcr_C03-1*	154	C03-V3_17,067,147	C03-V3_9,689,084	4.2	17.0	15.3	147.5	157.5

*QTL* quantitative trait loci; *LOD* logarithm of the odds; *PVE* phenotypic variation explained; *Add* additive; *CI* confidence interval

LOD, PVE(%), Add values and confidence interval (CI) from the estimated QTL position varied among the QTLs, ranging from 3.2 to 23.8 for LOD, 8.3–65.6% for PVE, 6.1–39.4 for Add and 1–13 cM for CI (Fig. 2, Table 2). Among these seven QTLs, two QTLs in *Rcr_C03-1* and *Rcr_C08-1* were identified to resistance to eight and two pathotypes, respectively, with higher LOD value (4.2–23.8 and 4.7–16.4), PVE (16.0–65.6 and 8.3–23.5%) and Add values (14.3–41.4 and 14.7–30.3) (Table 2). The values of Add for five QTLs (*Rcr_C01-1*, *Rcr_C03-1*, *Rcr_C03-2*, *Rcr_C03-3* and *Rcr_C08-1*) on C01, C03 and C08 were positive, indicating that the resistant loci were derived from the parent ECD11. However, QTLs (*Rcr_C04-1*, *Rcr_C08-2*) on C04, C08 were negative, indicating that the resistant loci were derived from the parent T010000DH3.

### Identification of potential candidates genes for the QTLs

Genes in the flanking region of the seven QTLs were identified using CDS of the reference genome sequence of ‘D134’ by Blast2GO (Supplementary Table S5-8) and searched for candidate genes that encoded disease resistance proteins and defense-related genes (Table 3, Supplementary Table S9).

**Table 3:** Disease resistant identified from gene ontology annotation using BLAST2GO in the flanking region of chromosome C01, C03, C08 in the *Brassica oleracea* reference genome sequence of cabbage doubled-haploid line ‘D134’ and corresponding homolog of Arabidopsis

QTLs (Corresponding pathotypes)	Gene name	Start	End	Description from Blast2Go	Homolog in Arabidopsis	Description from TAIR
*Rcr_C01-1* (2B)	*Boc01g01060.1*	8,299,581	8,303,522	Inactive disease resistance protein RPS4-like	*AT4G19510.2*	Disease resistance protein (TIR-NBS-LRR class)
*Rcr_C03-1* (2B, 3H, 3O, 5C, 5G, 8J, 5K, 5L)	*Boc03g00784.1*	9,533,165	9,534,856	Disease resistance-like protein	*AT2G14080.1*	Disease resistance protein (TIR-NBS-LRR class)
	*Boc03g00822.1*	10,163,079	10,168,624	Disease resistance protein LAZ5-like isoform X1	*AT4G19510.1*	Disease resistance protein (TIR-NBS-LRR class)
	*Boc03g00825.1*	10,212,132	10,216,729	Disease resistance-like protein CSA1	*AT4G19510.1*	Disease resistance protein (TIR-NBS-LRR class)
	*Boc03g00965.1*	11,746,815	11,747,411	Disease resistance protein RRS1 isoform X3	*AT4G36140.1*	Disease resistance protein (TIR-NBS-LRR class)
	*Boc03g00966.1*	11,747,534	11,747,845	Disease resistance protein RRS1 isoform X1	*AT4G36140.1*	Disease resistance protein (TIR-NBS-LRR class)
	*Boc03g00967.1*	11,747,913	11,748,485	Disease resistance protein RRS1 isoform X1	*AT4G36140.1*	Disease resistance protein (TIR-NBS-LRR class)
	*Boc03g00968.1*	11,748,756	11,749,391	Disease resistance protein RRS1 isoform X1	*AT4G36140.1*	Disease resistance protein (TIR-NBS-LRR class)
	*Boc03g00969.1*	11,749,509	11,749,942	Disease resistance protein RRS1 isoform X1	*AT4G36140.1*	Disease resistance protein (TIR-NBS-LRR class)
	*Boc03g00970.1*	11,751,167	11,755,194	Disease resistance-like protein CSA1	*AT4G36150.1*	Disease resistance protein (TIR-NBS-LRR class) family
	*Boc03g01016.1*	12,152,885	12,168,584	Probable disease resistance protein RPP1	*AT3G44400.1*	Disease resistance protein (TIR-NBS-LRR class) family
*Rcr_C08-1* (8J, 5K)	*Boc08g03058.1*	23,486,420	23,486,905	Probable disease resistance protein At1g12290	*AT1G12290.1*	Disease resistance protein (CC-NBS-LRR class) family
	*Boc08g03059.1*	23,487,114	23,487,434	Disease resistance protein RPS5-like	*AT1G12220.2*	Disease resistance protein (CC-NBS-LRR class)
	*Boc08g03179.1*	24,623,210	24,624,463	Probable disease resistance RPP8-like protein 2 isoform X1	*AT1G53350.1*	Disease resistance protein (CC-NBS-LRR class) family
	*Boc08g03180.1*	24,625,065	24,626,401	Probable disease resistance RPP8-like protein 2 isoform X1	*AT1G53350.1*	Disease resistance protein (CC-NBS-LRR class) family

*RPS* Resistance to *Pseudomonas syringae*; *TIR-NBS-LRR* toll-interleukin-1 receptor (TIR)- nucleotide-binding site- leucine-rich repeat; *LAZ* lazarus; *CSA* constitutive shade-avoidance; *RRS* resistance to *Ralstonia solanacearum*; *RPP* Recognition of *Peronospora parasitica*; *CC-NBS-LRR* coiled-coil-nucleotide-binding site-leucine-rich repeat

*Rcr_C01-1* derived from T010000DH3 was identified in the flanking region of 7.49–8.39 Mb of chromosome C01 for resistance to pathotype 2B. One hundred eleven genes were identified in the flanking region of *Rcr_C01-1*, where 7 genes (*Boc01g01056.1*, *Boc01g01060.1, Boc01g01022.1*, *Boc01g01023.1*, *Boc01g01024.1*, *Boc01g01063.1, Boc01g01078.1*) encoded proteins with function related to plant defense response (Supplementary Table S5). *Boc01g01060.1* is homologous to the Arabidopsis gene *AT4G19510.2*, which encoded TNL class protein, identified as possible candidate gene for *Rcr_C01-1* (Table 3, Supplementary Table S9).

*Rcr_C03-1* derived from ECD11 was identified in chromosome C03 in the flanking region of 9.21–17.06 Mb for resistance to 8 pathotypes (2B, 3H, 3O, 5C, 5G, 5K, 5L and 8J). Six hundred ninety genes were identified in the flanking region of *Rcr_C03-1*, where 10 genes (*Boc03g00784.1*, *Boc03g00822.1*, *Boc03g00825.1*, *Boc03g00965.1*, *Boc03g00966.1*, *Boc03g00967.1*, *Boc03g00968.1*, *Boc03g00969.1*, *Boc03g00970.1* and *Boc03g01016.1*) encoded TNL disease resistance proteins (Supplementary Table S6). *Boc03g00784.1* was homologous to the Arabidopsis gene *AT2G14080.1*. Two genes *Boc03g00822.1* and *Boc03g00825.1* were homologous to the Arabidopsis gene *AT4G19510.1*. Five genes *Boc03g00965.1*, *Boc03g00966.1*, *Boc03g00967.1*, *Boc03g00968.1* and *Boc03g00969.1* were homologous to the Arabidopsis gene *AT4G36140.1*. *Boc03g00970.1* is homologous to the Arabidopsis gene *AT4G36150.1*. *Boc03g01016.1* is homologous to the Arabidopsis gene *AT3G44400.1* (Supplementary Table S9). These ten *B. oleracea* genes homologous to Arabidopsis genes encoding TNL class proteins could be the candidates for *Rcr_C03-1*.

*Rcr_C03-2* derived from ECD11 was identified in chromosome C03 in the flanking region of 5.85–10.26 Mb for resistance to pathotype 3D. Forty-two genes were identified in the flanking region of *Rcr_C03-2*, where no gene encoded disease resistance proteins or function related to plant defense response (Supplementary Table S6).

*Rcr_C03-3* derived from ECD11 was identified in chromosome C03 in the flanking region of 35.22–36.28 Mb, for resistance to pathotype 3A. Eighty-eight genes were identified in the flanking region of *Rcr_C03-2*, where no gene encoded disease resistance proteins or function related to plant defense response (Supplementary Table S6).

*Rcr_C04-1* derived from T010000DH3 was identified in chromosome C04 in the flanking region of 51.28–52.14 Mb for resistance to pathotype 3D. Seventy-three genes were identified in the flanking region of *Rcr_C04-1*, where no gene encoded disease resistance protein or function related to plant defense response (Supplementary Table S7).

*Rcr_C08-1* derived from ECD11 was identified in chromosome C08 in the flanking region of 23.35–24.78 Mb for resistance to pathotype 5K and 8J. Ninety-nine genes were identified in the flanking region of *Rcr_C08-1*, where 4 genes (*Boc08g03058.1, Boc08g03059.1, Boc08g03179.1* and *Boc08g03180.1*) encoded disease resistance protein (Supplementary Table S8). *Boc08g03058.1* and *Boc08g03059.1* are homologous to Arabidopsis genes *AT1G12290.1* and *AT1G12220.2,* both genes encoded CNL class proteins (Table 3, Supplementary Table S9). Another two genes *Boc08g03179.1* and *Boc08g03180.1* are homologous to Arabidopsis gene *AT1G53350.1*, which also encoded CNL class proteins (Table 3, Supplementary Table S9). These four *B. oleracea* genes *Boc08g03058.1*, *Boc08g03059.1*, *Boc08g03179.1* and *Boc08g03180.1* homologous to Arabidopsis genes *AT1G12290.1*, *AT1G12220.2*, *AT1G53350.1* and *AT1G53350.1*, respectively, which encode CNL class proteins, could be possible candidate genes for *Rcr_C08-1*.

*Rcr_C08-2* derived from T010000DH3 was identified in chromosome C08 in the flanking region of 28.50–29.07 Mb for resistance to pathotype 5K. Thirty-six genes were identified in the flanking region of *Rcr_C08-2*, where no gene encoded disease resistance protein or function related to plant defense response (Supplementary Table S8).

Polymorphic sequence variants identified in the QTLs flanking region between the two parents of chromosome C01, C03, C04 and C08 were searched. Out of 1139 genes in the flanking regions only 52 genes produced polymorphic variants (Supplementary Table S10).

### Search for the syntenic regions of the two significant QTLs of C03 and C08 in the *B. rapa* ‘Chiifu’ reference genome sequence

*B. rapa* is a major source of CR genes or QTLs for clubroot disease resistance in *Brassica* species. In this study, the BC_1_ population was developed from *B. oleracea* with seven QTLs identified. More importantly, two QTLs *Rcr_C03-1* and *Rcr_C08-1* were identified for resistance to 8 and 2 pathotypes, respectively. *B. oleracea* chromosomes C03 and C08 have higher synteny with *B. rapa* chromosomes A03 and A08, respectively (Perumal et al. 2020). Many CR genes have been identified in chromosomes A03 and A08 (Yu et al. 2021; Rahaman et al. 2022). Two QTLs *Rcr_C03-1* and *Rcr_C08-1* with TNL or CNL genes identified in this study were compared with A03 and A08 of *B. rapa* using *B. rapa* reference genome sequence ‘Chiifu’ v3.0 (Zhang et al. 2018).

There were 10 TNL genes in the *Rcr_C03-1* regions of C03, nine genes were homologous to A03 genes of *B. rapa* ‘Chiifu’ v3.0 (Supplementary Table S9, S11). *Boc03g00784.1* (9,533,165–9,534,856), *Boc03g00822.1* (10,163,079–10,168,624), *Boc03g00825.1* (10,212,132–10,216,729), *Boc03g00965.1* (11,746,815–11,747,411), *Boc03g00967.1* (11,747,913–11,748,485), *Boc03g00968.1* (11,748,756–11,749,391), *Boc03g00969.1* (11,749,509–11,749,942), *Boc03g00970.1* (11,751,167–11,755,194) and *Boc03g01016.1* (12,152,885–12,168,584) were homologous to *B. rapa* A03 genes *BraA03g049830.3C* (25,366,429–25,366,902), *BraA03g048820.3C* (24,717,736–24,720,078), *BraA03g048820.3C* (24,717,736–24,720,078), *BraA03g056630.3C* (29,584,377–29,585,819), *BraA03g048830.3C* (24,729,618–24,732,164), *BraA03g016950.3C* (7,879,277–7,891,060) *BraA03g051100.3C* (26,191,764–26,194,343), *BraA03g017700.3C* (8,271,506–8,271,700), and *BraA03g009600.3C* (4,126,964- 4,129,030), respectively. There were 4 CNL genes identified in the *Rcr_C08-1* regions of C08 (Table S9, S11). *Boc08g03058.1* (23,486,420–23,486,905) and *Boc08g03059.1* (23,487,114–23,487,434) were homologous to a single *B. rapa* A08 gene *BraA08g031100.3C* (21,138,657–21,138,929), while *Boc08g03179.1* (24,623,210–24,624,463) and *Boc08g03180.1* (24,625,065–24,626,401) were homologous to a single *B. rapa* A08 gene *BraA08g032140.3C* (21,640,250–21,641,322). Seventy-three SNP markers identified in the flanking region of C01, C03 and C08 of *B. oleracea* reference genome sequence of cabbage DH line D134, which can be used for marker-assisted selection (Table S12).

## Discussion

Two major QTLs, *Rcr_C03-1* and *Rcr_C08-1*, and five minor QTLs, *Rcr_C01-1, Rcr_C03-2, Rcr_C03-3, Rcr_C04-1* and *Rcr_C08-2*, were detected from the current study on four chromosomes C01, C03, C04 and C08 against 10 pathotypes; 3A, 2B, 5C, 3D, 5G, 3H, 8J, 5K, 5L and 3O. Previous QTLs against different pathotypes were identified from different *B. oleracea* resistant sources on those chromosomes of C01, C03, C04 and C08 (Voorrips et al. 1997; Moriguchi et al. 1999; Rocherieux et al. 2004; Nagaoka et al. 2010; Lee et al. 2016; Peng et al. 2018). Farid et al. 2020 also identified QTLs on C03, C04 and C08 with two pathotypes, 3A and 5X-LG2 through the association mapping. In this study, ten pathotypes were used for identification of QTLs in *B. oleracea* through the bi-parental mapping method. As there was no common reference genome sequence and molecular markers that can be used for the identification of QTLs, it was not possible to find relationships of the QTLs identified in this study with those previously identified by others.

Due to the rapid emergence of new pathotypes and the A-genome CR resistance breakdown in 1st generation CR cultivars, it becomes an urgency to utilize polygenic resistance of *B. oleracea*. Until now, most of the CR genes identified in Canada from A-genome of *B. rapa* (Yu et al. 2017, 2016, 2021; Chu et al. 2014; Huang et al. 2017; Gao et al. 2014; Karim et al. 2020; Rahaman et al. 2022) and *B. napus* (Fredua-Agyeman et al. 2016; Hasan et al. 2016; Zhang et al. 2016). One gene on chromosome A03 was used for developing the 1st generation CR cultivars in Canada. Recently, a report has been published on CR resistance of C-genome for resistance to pathotype 3 and 5X-LG2 (Dakouri et al. 2018). The study was based on RNA-seq. Compared to RNA-seq, QTL mapping from BC_1_/BC_1_S_1_ lines could be used for identifying CR loci for multiple pathotypes effectively (Yu et al. 2017). Therefore, identification of CR resistance from C-genome with more pathotypes became a priority to expand *B. napus* CR resistance from C-genome.

In this study, we performed bi-parental QTL mapping with polymorphic SNPs through GBS from 124 BC_1_ lines using a latest reference genome sequence ‘D134’. Depending on different morphotypes in *B. oleracea* species, five reference assemblies were published (Parkin et al. 2014; Liu et al. 2014; Sun et al. 2019; Belser et al. 2018; Lv et al. 2020) as variability between different morphotypes is high. Two types of DNA sequencing technologies were used: short-read technology such as next-generation sequencing (NGS) and long-read technology such as Oxford Nanopore Technology (ONT) and Pacific Biosciences (PacBio). Two *B. oleracea* genome sequences available based on NGS technology: the TO1000 (kale-like; *B. oleracea* var. *alboglabra*) assembly (Parkin et al. 2014) and the 02–12 (cabbage; *B. oleracea* var. *capitata*) assembly (Liu et al. 2014), but their errors and gaps make them difficult to use for many studies (Lee et al. 2016; Liu et al. 2016, 2017; Zhang et al. 2015). Another three *B. oleracea* genomes recently published based on combination of short and long-read technologies; the C-8 (cauliflower; *B. oleracea* L. var. *botrytis*) assembly (Sun et al. 2019), the HDEM (Broccoli; *B*. *oleracea* L. var. *italica*) assembly (Belser et al. 2018) and the ‘D134’ (Cabbage; *B. oleracea* var. *capitata*) assembly (Lv et al. 2020). Long-read technology with using efficient algorithms can provide high-quality assemblies in chromosome-level (Jiao et al. 2017; Belser et al. 2018; Michael et al. 2018). As the CR parental line ECD11 is a cabbage cultivar, we used ‘D134’ reference genome sequence (Lv et al. 2020) as the template for SNP calling in this study.

The number of SNPs site per chromosome is often correlated with chromosome size (Yu et al. 2016). In this study, the number of SNPs and polymorphic SNPs identified in the population were strongly correlated (*R*^*2*^ = 0.84, 0.71) of the reference genome sequence (Table S3). The total numbers of SNPs shows a better correlation with the chromosome length than the number of polymorphic SNPs, suggesting that polymorphic SNPs may be clustered. However, this may not be an issue for filtering, construction of genetic map and subsequent QTL mapping as BIN function was performed to remove redundant SNP loci using IciMapping. Although polymorphic SNPs seem more clustered but still total number SNPs and polymorphic SNPs were also positively correlated (*R*^*2*^ = 0.65). Genetic linkage map is pre-requisite for QTL analysis and genetic mapping. Linkage map length could depend on population type and size, reference genome sequence size as well as number of polymorphic variants used for mapping. In this study, we constructed the linkage map with polymorphic variants of 124 BC_1_ lines using the latest reference genome sequence ‘D134’. It was 1,256.5 cM in length, longer than previously published *B. oleracea* linkage maps; 879.9 cM (Lee et al. 2016), 913.5 cM (Haque et al. 2017) and 1028.0 cM (Peng et al. 2018).

The R parental line ECD11 was moderate to highly resistant to all 10 pathotypes, while S parental line T010000DH3 was highly susceptible for all pathotypes. The F_1_ plants from the interspecific crosses T010000DH3 × ECD11 were moderate to highly susceptible to all pathotypes and BC_1_S_1_ population showed continuous distribution of %DSI (Fig. 1), which indicate that CR resistance of these 10 pathotypes is controlled by QTLs. Similar quantitative resistance was reported in *B. oleracea* for clubroot (Figdore et al. 1993; Nagaoka et al. 2010) as well black rot (Tonu et al. 2013) diseases. The frequency distribution in the BC_1_S_1_ population for resistance to some pathotypes also showed skewed tendency, similar skewed phenotype also reported for clubroot quantitative resistance in *B. oleracea* (Figdore et al. 1993; Peng et al. 2018; Ce et al. 2021). These results indicated that ECD11 clubroot resistance is controlled by quantitative manner with multiple genes effect.

A QTL that can be consistently detected with a PVE of > 10% of trait value can be designated as the main effect QTL or major QTL (Wang et al. 2019). In this study, one QTL, *Rcr_C03-1* on C03 which contributed 16.0–65.6% phenotypic variation for 8 pathotypes; 2B, 5C, 5G, 3H, 8J, 5K, 5L and 3O (Table 2). Another QTL, *Rcr_C08-1* were identified with 8.3 and 23.5% PVE for resistance to 8J and 5K, respectively (Table 2). As these two QTLs were identified for resistance to more than one pathotypes with PVEs of > 10% in the most cases. They can consider as major QTLs in this study. These two QTLs in *Rcr_C03-1* and *Rcr_C08-1* were identified with higher LOD value, PVE and Add values, indicating that they might have significant contribution for CR resistance in ECD11. Previously, one major QTL (*Pb-Bo1*) was detected on C01 for five isolates (Pathotypes 1, 2, 4 & 7; according to the classification by Some et al. 1996) and explained 20.7–80.7% of the phenotypic variation (Rocherieux et al. 2004). Five minor QTLs *Rcr_C01-1, Rcr_C03-2, Rcr_C03-3, Rcr_C04-1* and *Rcr_C08-2* were detected on chromosomes C01, C03, C04 and C08 which contributed 8.3–18.7% PVE for only one pathotype each (Table 2). A previous study reported that a single involvement of the major CR gene, or accumulation of CR genes in the minor CR–QTL, is not enough to confer sufficient resistance (Tomita et al. 2013). Further investigations for understanding individual or cumulative effect of identified major QTLs in ECD11 are needed. The values of Add for the five QTLs (*Rcr_C01-1, Rcr_C03-1, Rcr_C03-2, Rcr_C03-3, Rcr_C08-1*) on C01, C03 and C08 were positive, while for 2 QTLs (*Rcr_C04-1* and *Rcr_C08-2*) on C04 and C08 were negative, indicating that the five resistant loci were derived from the resistant parent ECD11 and two resistant loci were derived from the susceptible parent T010000DH3. The presence of QTL for resistance to clubroot in T010000DH3 needs further confirmation. Previously, a resistant locus, *PbBo(GC)1* identified from susceptible broccoli parent account for 9% of the phenotypic variation (Nagaoka et al. 2010).

Among identified 7 CR QTLs, disease resistance proteins and defense-related genes were identified in three QTLs (*Rcr_C01-1*, *Rcr_C03-1* and *Rcr_C08-1*) (Table 3). From the *Rcr_C01-1* flanking region, a potential candidate TNL gene *Boc01g01060.1* was identified. From the *Rcr_C03-1* flanking region, ten TNL genes (*Boc03g00784.1*, *Boc03g00822.1*, *Boc03g00825.1*, *Boc03g00965.1*, *Boc03g00966.1*, *Boc03g00967.1*, *Boc03g00968.1*, *Boc03g00969.1*, *Boc03g00970.1* and *Boc03g01016.1*) encoding disease resistance proteins were identified, which were homologous to five Arabidopsis genes. Two genes *Boc03g00822.1* and *Boc03g00825.1* were homologous to *AT4G19510.1* and five genes *Boc03g00965.1*, *Boc03g00966.1*, *Boc03g00967.1*, *Boc03g00968.1* and *Boc03g00969.1* were homologous to *AT4G36140.1*. Identified candidate CR genes of *B. oleracea* which are homologous to the same Arabidopsis gene producing similar resistance proteins or not are to be determined. From the *Rcr_C08-1* flanking region, 4 genes (*Boc08g03058.1, Boc08g03059.1, Boc08g03179.1* and *Boc08g03180.1*) encoding disease resistance proteins were identified, which were homologous to 3 Arabidopsis genes and produce CNL class proteins. TNL genes are rare in monocotyledons, but CNL genes are quite common in both monocotyledons and dicotyledons (Tarr et al. 2009; Liu et al. 2021). Although previously three genes *CRa, Crr1a* and *CRb* have been cloned and TNL class proteins were reported for clubroot resistance from dicot Brassica. (Ueno et al. 2012; Hatakeyama et al. 2013, 2017), there is no report on CNL class proteins for resistance to clubroot.

In this study, CR QTLs with disease resistant genes were identified in chromosomes C01, C03 and C08 from ECD11. *B. oleracea* chromosomes C03 and C08 have higher synteny with *B. rapa* chromosome A03 and A08 (Perumal et al. 2020). In addition, A03 and A08 contain many CR genes. Therefore, the identified major QTLs of C03 and C08 with candidate disease resistant genes in this study were compared with A03 and A08 of the *B. rapa* reference genome sequence ‘Chiifu’ v3.0 (Zhang et al. 2018) (Supplementary Table S11). For *Rcr_C03-1*, among ten candidate genes of C03, four genes, *Boc03g00784.1*, *Boc03g00822.1*, *Boc03g00825.1* and *Boc03g00967.1*, were homologous to *B. rapa* A03 genes *BraA03g049830.3C* (25,366,429–25,366,902), *BraA03g048820.3C* (24,717,736–24,720,078), *BraA03g048820.3C* (24,717,736–24,720,078) and *BraA03g048830.3C* (24,729,618–24,732,164), which resides in the flanking region of previously identified CR major gene *Rcr1* (24.57–25.71 Mb) and *Rcr4* region (23.82–26.08 Mb). One gene, *Boc03g00969.1*, was homologous to *B. rapa* A03 gene *BraA03g051100.3C* (26,191,764–26,194,343), resides in the flanking region of previously identified QTL, *Rcr4* region (23.82–26.08 Mb). Rest of the four genes, *Boc03g00965.1*, *Boc03g00968.1*, *Boc03g00970.1* and *Boc03g01016.1*, were homologous to *B. rapa* A03 genes *BraA03g056630.3C* (29,584,377–29,585,819), *BraA03g016950.3C* (7,879,277–7,891,060), *BraA03g017700.3C* (8,271,506–8,271,700) and *BraA03g009600.3C* (4,126,964–4,129,030) accordingly. According to our knowledge, no CR genes were reported previously for these four genes, so these four genes could be considered as novel candidate genes in the cluster for CR resistance. For *Rcr_C08-1*, four candidate resistant genes were identified. *Boc08g03058.1* and *Boc08g03059.1* were homologous to a single *B. rapa* A08 gene *BraA08g031100.3C* (21,138,657–21,138,929), while *Boc08g03179.1* and *Boc08g03180.1* were homologous to a single *B. rapa* A08 gene *BraA08g032140.3C* (21,640,250–21,641,322). Polymorphic sequence variants in the QTLs flanking regions were also searched between the two parents (Supplementary Table S10). Out of 1,139 genes in the flanking region only 52 genes produced polymorphic variants. Even though higher coverage of GBS in the parents, only two defense-related genes (*Boc01g01078.1* and *Boc03g00965.1*) produced polymorphic variants. Although the genome sequence of susceptible line DH3 is available, whole genome sequencing of resistant line ECD11 is needed so comparison of two genome sequences in the target regions could be performed. Cloning of the genes is also required for confirming the CNL gene involvement for CR resistance. This study focuses on identification of QTLs and mapping QTLs into the respective genomic regions. There are genes encoding disease resistance proteins in some of the QTL intervals. However, further study will be needed to determine if they confer tolerance or resistance. The comparison of previously identified QTLs on C01, C03, C08 was not possible due to lack of common molecular markers, reference genome and different source of clubroot pathogen pathotypes used in those studies. A total 15 genes encoding TNL/CNL class disease resistance proteins were identified in the *Rcr_C01-1*, *Rcr_C03-1* and *Rcr_C08-1* flanking regions for resistance to 10 pathotypes. Identified CR QTLs from *B. oleracea* cultivar ECD11 can be transferred to *B. napus* either interspecific breeding or developing new re-synthesized *B. napus*, which could significantly contribute to development of new CR canola varieties against new races of *P. brassicae* in Western Canada.

### Supplementary Information

Below is the link to the electronic supplementary material.

Supplementary file1 (XLSX 313 KB)

Evaluation of plants for resistance to clubroot using a 0 – 3 scale (PNG 742 KB)

The linkage map of* Brassica oleracea* consisting of 1,414 SNP sites extracted from using* Brassica oleracea* reference genome sequence of cabbage doubled haploid line ‘D134’ (PNG 98 KB)

## Data Availability

The raw datasets generated and/or analyzed during the current study are available in the National Center for Biotechnology Information (NCBI) repository, BioProject accession: PRJNA948018. Data will be released on 31 July, 2023, or upon manuscript publication. https://www.ncbi.nlm.nih.gov/sra/PRJNA948018.
