# Supplementary figures and images for: Identification of QTLs for resistance to 10 pathotypes of Plasmodiophora brassicae in Brassica oleracea cultivar ECD11 through genotyping-by-sequencing
Source: Theor Appl Genet. 2023 Nov 20;136(12):249. doi: 10.1007/s00122-023-04483-y (PMC10661809; doi:10.1007/s00122-023-04483-y)

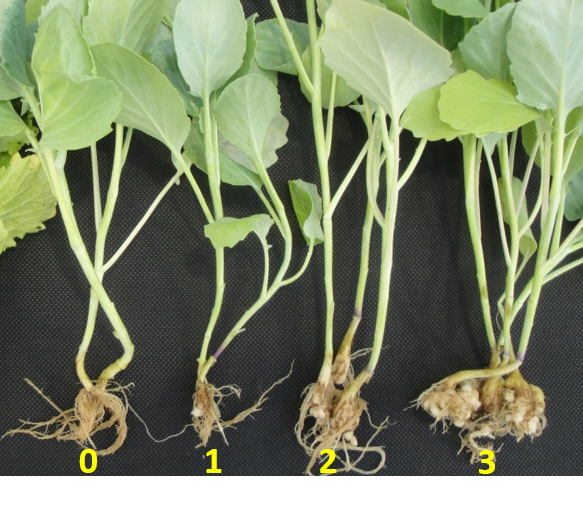

Supplement: Supplementary file 2 — Evaluation of plants for resistance to clubroot using a 0 – 3 scale (PNG 742 KB) [file 122_2023_4483_MOESM2_ESM.png]

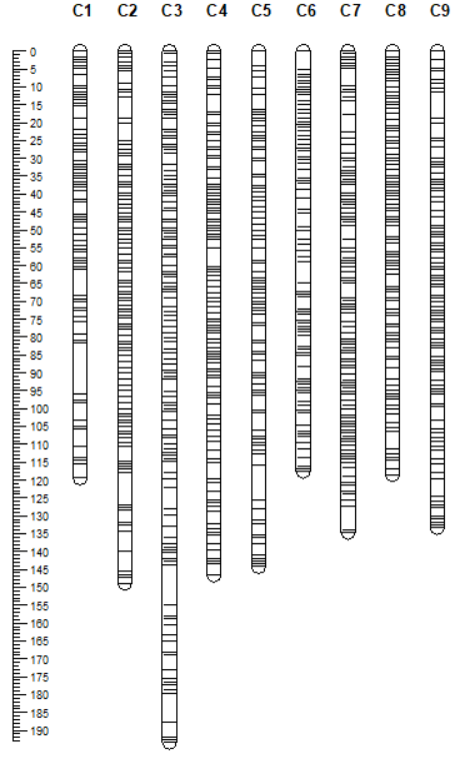

Supplement: Supplementary file 3 — The linkage map of Brassica oleracea consisting of 1,414 SNP sites extracted from using Brassica oleracea reference genome sequence of cabbage doubled haploid line ‘D134’ (PNG 98 KB) [file 122_2023_4483_MOESM3_ESM.png]
